# Leptin physiology and pathophysiology: knowns and unknowns 30 years after its discovery

**DOI:** 10.1172/JCI174595

**Published:** 2024-01-02

**Authors:** Jeffrey S. Flier, Rexford S. Ahima

**Affiliations:** 1 Department of Medicine and Neurobiology, Harvard Medical School, Boston, Massachusetts, USA.; 2 Division of Endocrinology, Diabetes, and Metabolism, Department of Medicine, Johns Hopkins University School of Medicine, Baltimore, Maryland, USA.

## Discovery of leptin

The cloning of the *ob* gene in 1994 and reports in 1995 that administration of the encoded protein leptin reversed obesity of *ob/ob* mice that lacked it were major breakthroughs in endocrinology and metabolism (1, 2). The name leptin was derived from leptos, the Greek root for thin, because leptin was initially considered to be a signal from adipose tissue to the brain; rising levels of leptin acted through a negative feedback mechanism to limit obesity by reducing food intake and increasing energy expenditure.

The discovery of leptin did not occur in a vacuum. Before 1994, substantial research in rodents and humans provided evidence of homeostatically regulated responses that acted to resist body weight induced by overfeeding or underfeeding (3). Experiments employing hypothalamic lesions as well as a joining of the circulation between rodents (parabiosis) suggested that one or more circulating factors informed the brain of energy stores (4). However, the identity of such factors was unknown, as was their tissue of origin, mode of regulation, and how they effected physiologic responses.

The discovery that leptin was an adipose-derived hormone that informed brain targets of energy stores was a foundational centerpiece of modern metabolic science. But emerging leptin biology has produced many surprises, and important questions remain unanswered. This Viewpoint is an assessment of current understanding, stressing insights needed to advance the field.

## Uncovering the role of leptin in obesity

Complete absence of leptin or its inability to signal, caused by loss-of-function mutations of the ligand or its receptor in both rodents and humans, respectively, produces profound obesity. Consistent with classic endocrine logic, leptin replacement reverses obesity due to leptin mutations but does not correct obesity caused by mutations in its receptor. Contrary to the initial idea of leptin being an “antiobesity hormone,” avoidance of obesity is not leptin’s dominant physiologic role. Leptin expression and circulating levels increase and reflect the degree of adiposity in diet-induced obese and several mouse obesity models, but hyperleptinemia clearly doesn’t prevent obesity (5). Hyperleptinemia proportional to obesity was also observed in humans. Thus, leptin resistance appears to be present in most cases of obesity, perhaps analogous to insulin resistance in type 2 diabetes.

Obesity treatment is an enormous unmet medical need for which leptin initially appeared a logical answer, and development of leptin for treatment of obesity was aggressively pursued. However, unlike the benefit of supplemental insulin treatment in individuals with diabetes with insulin resistance, leptin treatment had little or no effect to reduce obesity in the general population, and thus the strategy was quickly abandoned (6).

Simultaneously, another distinct physiologic role for leptin was emerging: the ability of falling leptin levels to signal the *starved state* to the brain. The rapid fall of leptin expression and levels with starvation was first observed in 1995 in mice (7). In 1996, falling leptin was shown to be necessary and sufficient to cause the neuroendocrine adaptation to starvation — including the suppression of reproduction (8). Leptin administration to prepubertal female mice has a permissive action in accelerating the onset of puberty, further highlighting the important linkage between leptin and neuroendocrine function, and repletion of leptin restored menstrual cycles in women with hypoleptinemic amenorrhea (9, 10). Taken together, it is now established that leptin is an adipocyte-derived hormonal signal to the brain that drives the transition between the physiology of starvation and energy sufficiency.

So, what is leptin’s role in “common obesity”? Why is hyperleptinemia unable to prevent weight gain in most people? This topic has generated substantial confusion and, of late, has been largely ignored. We address some of the reasons here, beginning with our understanding of obesity and leptin in rodents. C57BL/6 mice fed a diet high in fat and sugar develop obesity (called diet-induced obesity [DIO]); these mice are now widely seen as the best (albeit imperfect) rodent model for common human obesity. Leptin expression and levels rise as obesity develops in DIO mice, and leptin administration has little (or no) effect to reverse obesity, suggesting “resistance” to endogenous and exogenous leptin (11).

It is important to stress that this leptin resistance in DIO mice is only partial, as obesity in these mice is much less severe than obesity in *db/db* mice completely lacking leptin receptor. Some leptin signals are still being sensed in DIO mice — including the brain signal to maintain reproductive competence.

What causes this partial leptin resistance? A key early approach was to search for an antagonist of leptin signaling in hypothalamic target cells after acute leptin administration. The first and best studied such molecule was suppressor of cytokine signaling 3 (SOCS3), an intracellular inhibitor of leptin signaling shown to be acutely induced by leptin in hypothalamic neurons and found to be increased in DIO mice (12). A role for SOCS3 in leptin resistance is also supported by genetic models, where haploinsufficiency of SOCS3 confers protection against DIO, as does SOCS3 knockout in leptin target neurons, such as those expressing pro-opiomelanocortin (POMC) (13, 14). Inaccessibility of the hypothalamus has made it impossible to determine whether SOCS3 expression in key target cells influences susceptibility to obesity in humans. Although SOCS3 is an attractive target for treatment of obesity, its general inhibition would likely have adverse effects because SOCS3 inhibits signaling by many cytokines.

Whatever its intracellular mechanism, does hyperleptinemia itself cause leptin resistance? In one mouse model of DIO, hyperleptinemia was found to be required for leptin resistance, an idea further supported by evidence that *lowering* leptin levels in DIO mice with an anti-leptin antibody improves energy homeostasis (15, 16). Further complexity regarding the role of leptin in energy feedback was uncovered in a model of mouse obesity caused by forced overfeeding, with leptin levels “clamped” at normal levels and unable to rise. When forced overfeeding is ended, spontaneous feeding was suppressed (reflecting negative feedback) to the same extent in mice without hyperleptinemia as in hyperleptinemic DIO mice (17). This finding suggested a signal *other than leptin* must have suppressed feeding in response to obesity. The identity of such a factor, its actions, and possible role in obesity pathogenesis remain unknown at present.

Importantly, sensitivity to DIO varies widely across mouse strains, with some, like male C57BL/6 mice, being highly sensitive to obesity and others being resistant (18, 19). The role of leptin, leptin resistance, and/or other factors in accounting for genetic differences in DIO susceptibility should be an important subject of future research.

The discovery of leptin stimulated an explosive expansion of research on the neural circuits that regulate energy homeostasis in response to leptin and other factors. Neurons in the arcuate nucleus expressing POMC and agouti-related peptide (AgRP)/neuropeptide Y (NPY) respond directly to leptin. Activating and inhibitory signals from POMC and AgRP/NPY neurons, respectively, converge on downstream neurons expressing melanocortin 4 receptors, whose activation suppresses appetite (20). The complexity of central neural circuitry and how it integrates leptin and other signals is a subject of intense research.

## Remaining gaps in knowledge

Apart from the severe genetic disruptions of the leptin pathway that led to its discovery, we know surprisingly little 30 years later about the role of leptin action and resistance in human obesity. This knowledge gap is highlighted by comparing insights into leptin biology with knowledge about the physiology and biochemistry of insulin action in type 2 diabetes. Innumerable studies have quantified the effects of insulin infusions at varying doses on insulin action (glucose uptake, hepatic glucose production, lipolysis, etc.) and signaling in target tissues, such as fat and muscle, which led to the emergence of major insights for the field. In stark contrast, over 30 years, virtually no such studies have been performed with leptin in lean and obese humans.

**Figure 1:**
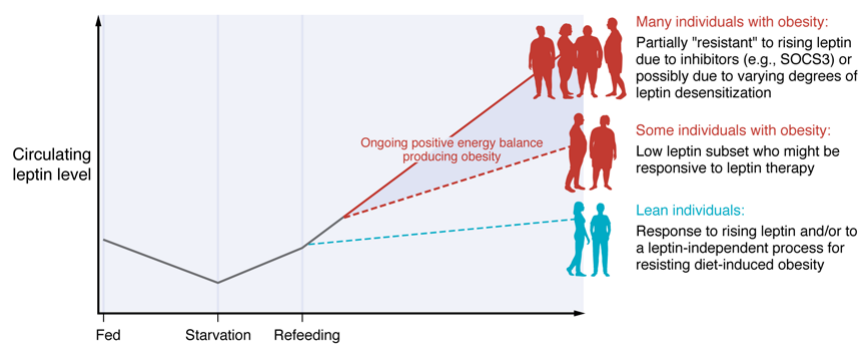
Patterns of leptin levels in response to changes in feeding and adiposity. Circulating leptin levels fall with starvation and rise to prior levels with refeeding. It is possible, but not yet demonstrated, that some lean individuals respond briskly to rising leptin to prevent obesity or respond to another as-yet undiscovered signal to prevent obesity. It is also possible, but not yet demonstrated, that some individuals with obesity with relatively low leptin levels for their degree of obesity might respond to exogenous leptin with weight loss. Most individuals with obesity have high leptin levels to which they are partially resistant, causing them to be unresponsive to exogenous leptin. This “leptin resistance” may be caused by desensitization induced by hyperleptinemia.

Which studies might be of greatest interest? Are individuals who remain lean (and healthy) without dieting or medications a subset with exquisite sensitivity to rising leptin, consistent with the initial hypothesis that leptin serves as an antiobesity “adipostatic” signal (Figure 1)? Similarly, are individuals with obesity with modest hyperleptinemia a subgroup that might be responsive to leptin therapy, with appetite suppression and reduction in fat mass in response to exogenous leptin (Figure 1)? These important questions could be answered by infusing leptin into such individuals (and relevant controls) and measuring the effects on hunger and food ingestion, among other responses.

Why haven’t such obvious human experiments been conducted? There are several possible answers. Perhaps, following the failure of leptin trials, investigators and the pharmaceutical industry lost interest in the topic and moved on to more appealing and tractable questions. This disinterest might have been furthered by the lack of easily quantifiable leptin responses, compared with insulin, where glucose is pertinent and easily measured. Inaccessibility of critical leptin target tissues for biochemical analysis is another obvious impediment. On the other hand, despite these issues, key insights into leptin physiology and resistance and their role in obesity would have been seen as important discoveries.

Perhaps the most likely explanation for the limited progress toward understanding leptin biology in human obesity is the unavailability of leptin for human clinical investigations. Three companies developed leptin analogs for potential use in obesity, and the failure of studies of these analogs to produce sufficient clinical benefit caused these efforts to be terminated. Along the way, internal studies and analyses that might have been done were never published, and requests from investigators for the hormone were typically denied. Rights to the best-studied analog, metreleptin, were passed to progressively smaller companies. Today, the sole use of leptin is for treatment of exceptionally rare cases of total leptin deficiency as well as rare lipodystrophies, where reversal of hypoleptinemia has beneficial metabolic effects (21).

Obesity is a major health crisis facing the US and other nations. Regrettably, research on one of the most important metabolic discoveries of the past 50 years has been limited by a lack of availability of recombinant leptin for human research. Scientific leaders and funders like NIH should understand the challenges and make a commitment to solving this problem. Only then might the great potential for leptin’s discovery to illuminate the pathophysiology and treatment of obesity be fulfilled.

## Version 1 01/02/2024

Electronic publication
